# Centralized Lung Nodule Management at A Veterans Hospital Using A Multidisciplinary Lung Nodule Evaluation Team (LNET)

**DOI:** 10.3779/j.issn.1009-3419.2018.11.04

**Published:** 2018-11-20

**Authors:** William R. WRIGHTSON, Umar GAUHAR, Fred HENDLER, Teresa JOINER, Jennifer PENDLETON

**Affiliations:** Robley Rex Veterans Affairs (VA) Medical Center, Louisville, Kentucky, USA

**Keywords:** Lung nodule, Cancer, Multidiscliplinary

## Abstract

**Introduction:**

Lung nodules are frequently identified on imaging studies and can represent early lung cancers. We instituted the Lung Nodule Evaluation Team (LNET) to optimize management of these nodules by a lung specialist physician. All lung nodules identified by a radiologist prompted a direct consultation to this service. We report our initial experience with this process.

**Methods:**

This is a retrospective review of patients with lung nodules at a single institution from 2008 to 2015. Since October 2014, lung nodules > 3 mm identified on computed tomography (CT) scanning of the chest generate an automatic consult to LNET from the radiology service. Demographic, nodule and follow up data was entered into a surveillance database and summarized.

**Results:**

There were 1, 873 patients identified in the database. Of these, 900 patients were undergoing active surveillance. Consults increased from 5.5 to 93 per month after the start of the new consult program. Lung nodules were identified on 64% of chest CT scans. Prior to the direct radiology consult the average size of a nodule was 1.7 cm and 0.7 cm after. The overall time from initial nodule imaging to initiating a management plan by a thoracic specialist physician was 3.7 days.

**Conclusion:**

Assessment of lung nodules by a specialist physician is important to ensure appropriate long term management and optimize utilization of diagnostic interventions. A direct radiology consult to a specialized team of chest physicians decreased the time in initiating a management plan, identified smaller nodules and may lead to a more judicious use of health care resources in the management of lung nodules.

## Introduction

Lung nodules are frequent incidental findings on computed tomography (CT) scans of the chest, requiring further follow up based on size, location, and patient background characteristics. Multiple protocols exist to provide follow up guidance, with the goal being to identify the 1%-5% which will ultimately be diagnosed as having malignant disease^[1-3]^. Our facility serves a catchment area that is endemic for both benign lung nodules and malignant disease. Kentucky has one of the highest mortality rates associated with lung cancer. Determining which nodules carry the highest risk can be difficult and a multidisciplinary approach may provide better outcomes. The National Comprehensive Cancer Network (NCCN) guidelines recommend a multidisciplinary evaluation of all lung nodules ≥3 mm in size insuring that a dedicated team of specialists are involved in nodule evaluation to apply the most current standards of care^[4]^.

Primary care physicians are frequently responsible for interpreting these lesions and determining optimal management. This often leads to unnecessary diagnostic imaging, invasive procedures and ultimately delays in providing appropriate definitive care. Ultimately, significant delays to definitive treatment are a missed opportunity for early intervention of lung cancer. In an effort to consolidate lung nodule management, we instituted the Lung Nodule Evaluation Team (LNET). This multidisciplinary group received direct consultation from the radiologist when a nodule was identified on chest computed tomography (CT) scans, potentially eliminating delay in referral to specialist care. We report our experience with this process.

## Methods

We retrospectively reviewed our LNET database of patients seen from November 1, 2008, to October 1, 2015. Patients included in this cohort had a lung nodule (solid, sub-solid, or ground glass opacity) identified on CT scanning at the Robley Rex VA Medical Center, and were actively managed thru our LNET system. All patients had a consult generated to LNET as a result of the presence of a nodule (solid or ground glass) ≥3 mm on CT scanning. Prior to October 2014, these consults were directly referred from primary care providers or other specialist services. After October 2014, a direct consult was automatically generated for all pulmonary nodules ≥3 mm identified on radiology reports of all CT scans done in the institution.

These patients were initially triaged to determine if they already had an assigned follow up with a specialist service or were currently enrolled in the database. Those patients without an assigned disposition were reviewed by a thoracic specialist (pulmonary medicine or thoracic surgeon). Determination is made to advance the patient for review by LNET in its weekly meeting or to assign the patient to an appropriate management algorithm.

Management algorithms included the following:

1. Surveillance with repeat imaging

2. Diagnostic intervention

3. Definitive disposition (Oncology, Thoracic Surgery, Pulmonary Medicine, Discharge)

Patients were entered into the database to ensure accurate and complete follow up. A nurse navigator contacted the patient either through the mail or by phone to notify them of the diagnostic or treatment plan. An informational flyer was distributed to the patient to educate them on lung nodules and surveillance process. The nurse navigator further served as a liaison between the patients and the multiple services involved in the patient's care. Monthly reports were generated from the database to identify patients due for surveillance follow up. These reports were reviewed by a thoracic specialist with the patients reassessed at each interval for continued surveillance, diagnostic studies or final disposition.

The multidisciplinary LNET conference met weekly to discuss challenging patients and to facilitate work up and appropriate management. The team consisted of thoracic surgery, pulmonary medicine, interventional pulmonary, medical oncology, and interventional radiology. Patients presenting with more complex nodules or for consideration of intervention were presented at the weekly meeting. Management decisions were made in the conference and implemented to facilitate diagnosis and treatment. Outcomes and quality measures were reviewed to aid in quality improvement.

## Results

From 2008 to 2015 database included totaled 1, 873 patients. Patients prior to the direct LNET consult system [October 2014 (*n*=877) and after (*n*=996)]. Patients with a current or prior smoking history represented 67% of the patients with nodules. Patient and nodule characteristics are shown in table 1.

**1:** Patient and nodule characteristics

Total patients	*n*=1, 873	Percent
Patients prior to LNET	877	47%
Patients after LNET	996	53%
Smoker	775	41%
Former smoker	496	26%
Never	288	15%
Unknown	314	17%
Location		
Left lung	723	39%
Upper	359	
Lower	364	
Right lung	1150	61%
Upper	469	
Middle	196	
Lower	485	
LNET: Lung Nodule Evaluation Team.		

### Consults and work load

The direct radiology consult resulted in a significant increase in LNET consultation from an average of 5.5 to 93 completed consults per month. Monthly surveillance review of the database generated an average of 132 patient films to review and reassess management decisions (Figure 1). This included patients completing their recommended follow up imaging as well as those who did not due to some form of non-compliance.

**1:**
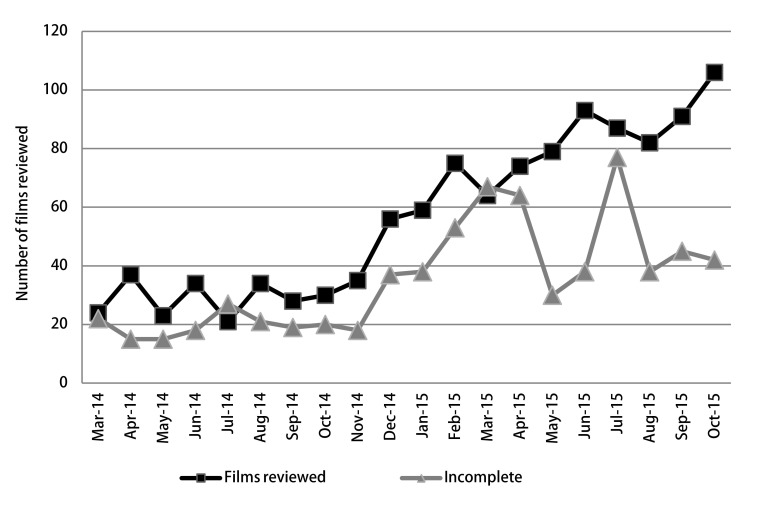
The chart shows the number of surveillance films reviewed for patients who are active in the database. Incomplete data point represents those patients who did not get films done on the date scheduled and required further contact or education. April 15 is the date the LNET Navigator was hired with a rapid decrease in non-compliance. The peak noted July 2015.

### Nodules identified on CT

Patients receiving CT scans of the chest had incidental nodules > 3 mm identified on 64% of the scans done at our facility. There was not a significant difference in the overall numbers of chest CT scans completed before versus after the directed consult system with an average of 320 films per month. Nodules were identified at a smaller size 0.7 cm after the directed consult versus 1.7 cm before. Lung nodules < 6 mm in size represented 45% of all the lung nodules identified (Table 2).

**2:** Lung nodule sizes identified in the current database

Size	Percent
< 6 mm	45%
6 mm-8 mm	24%
> 8 mm	31%

### Speed of specialist review

Once a nodule was identified, a consult was generated to LNET and reviewed by a thoracic specialist within an average of 3.4 days. Prior to the direct consultation system, this average was greater than 55 days (Figure 2). Patients were followed to a 2-year or greater time period in 21.5% of the cases. Patients were referred to surgery or interventional pulmonology for diagnostic interventions or evaluation in 7% and to Oncology in 8%. The majority of the unknown disposition represented those patients who were noncompliant with follow up recommendations (Table 3).

**2:**
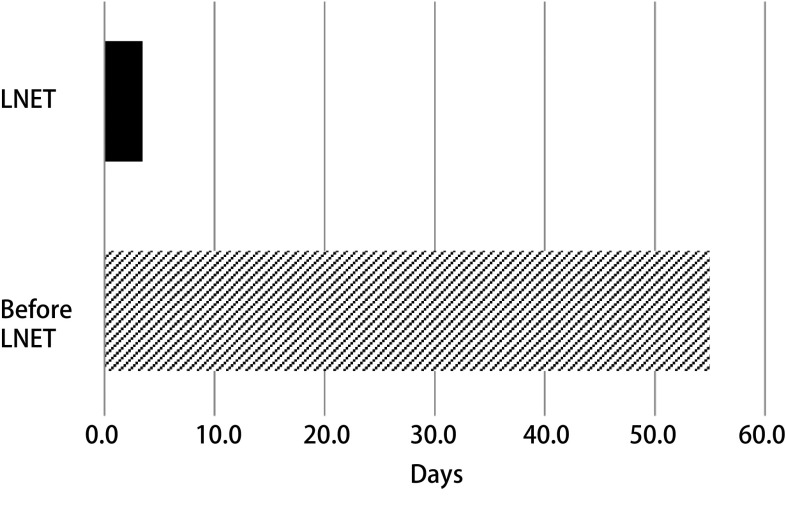
The average time from consultation to specialist evaluation decreased to 3.4 days. Represented a planned vacation for the newly hired navigator.

**3:** Patient disposition at discharge from database

Disposition	Percent	Patients
Stable > 2 years	23%	422
Resolution	7%	126
Oncology	7%	140
Surgery	7%	138
Death	5%	90
Transferred care	1%	13
Other	17%	323

## Discussion

Lung nodule management can be a challenging process, and consistency in follow-up and referral in a large healthcare system complicates this process. Mixed information and inconsistent application of multiple guidelines can lead to prolonged wait times, with excessive and often unnecessary imaging. Additionally, patient confusion and non-compliance may contribute to follow-up and delayed diagnosis. Our initial approach was to provide primary care physicians a system where they could easily refer patients for surveillance by a lung specialist (Thoracic Surgeon or Pulmonologist). However, we found that process of care failed to capture all patients with nodules and more importantly did not capture clearly suspicious nodules. In many instances, a > 8 mm spiculated nodule would go through expensive, incomplete and often times unnecessary invasive diagnostic studies prior to specialist consultation. Our change of process to allow for direct consultation to lung specialists captured the majority of lung nodule pathology in our institution with the result of more effective and efficient surveillance and diagnostic interventions.

Wait times from identification of a nodule to instituting an effective plan have decreased since instituting the LNET program. Reported wait times from identification to therapeutic intervention can be excessive due to multiple obstacles. The VA system reported wait times for instituting therapy at 71 days-90 days^[5-7]^. These most significant contributor to these wait times is how long it takes a primary care provider to consult a specialist and if they pursue their own diagnostic protocols prior to consultation (Figure 3). We have shown that at least one element of this process can be decreased by getting the patient to a lung specialist early on to institute a management plan, and in our institution that timeframe is currently 3.4 days. While this study does not include times to treatment of identified lung cancers, it does show a rapid progression from identification of a nodule to institution of a management plan.

**3:**
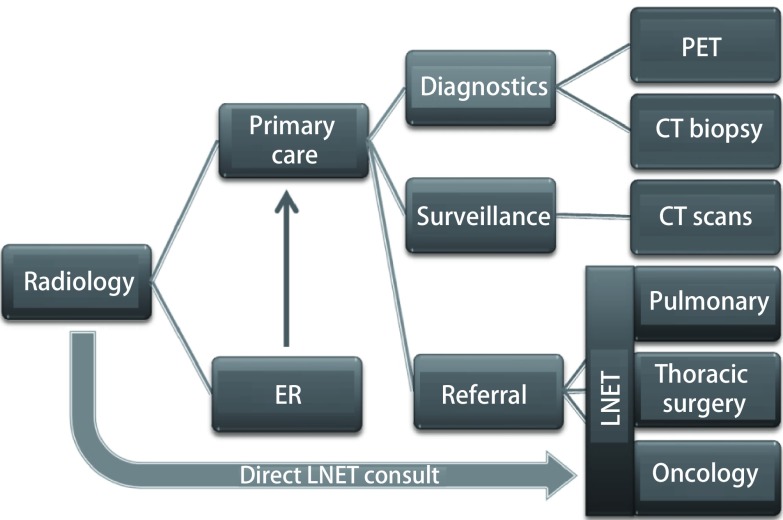
The radiologist is the only consultant in the chart that evaluates all the lung nodules in a system. By utilizing a direct consult from the radiologist to LNET, delays are minimized with an efficient utilization of resources.

One difficulty is the process of educating patients and guiding them thru the process. We have found a dedicated nurse navigator whose sole work assignment is within LNET to be invaluable. The nurse navigator assists in providing education, scheduling surveillance scans, and functions as a liaison between diagnostic and therapeutic services. The LNET nurse navigator ensures that studies are completed as scheduled and that patients are not lost to follow-up.

The majority of non-compliance was attributed to insufficient patient education regarding nodules and their potential significance. Other factors possibly include CT scheduling difficulty, lack of transportation to the hospital, and patients electing not following the specialist recommendation. The addition of a nurse navigator to better inform the patients of their diagnosis and facilitate scheduling CT scans reduced this rate from 42% to 28%.

The LNET consultation process has resulted in the identification of significantly smaller index nodules. The value of this is open to speculation. It could be that this identifies a subset of patients with smaller lesions that are more likely to be benign. While most studies suggest a relatively low risk of malignancy in nodules < 6 mm, that risk increases with increasing size. Currently, our average initial nodule size is 7 mm, compared to 17 mm previous to the direct radiology consult. There are several factors leading to the detection of smaller nodules. Nearly half of the incidental nodules identified were < 6 mm and these accounted for the largest increase in volume of consultation. One reason for instituting the current system was that a LNET consult was not generated until the primary care physician felt it was clinically indicated or the radiologist recommended specialist referral. We believe clinically significant nodules (> 6 mm) frequently were not being referred for specialist evaluation. Of note, there was a 69% increase in clinically significant nodules (> 6 mm) referred after the direct consultation started.

Identification of smaller nodules and early specialist consultation may lend itself to more appropriate resource utilization and detection of earlier cancers. In addition, the new Fleischner guidelines have been introduced that will eliminate those nodules < 6 mm in size from routine follow up^[8]^. Based on our data, this will eliminate 45% of the currently followed nodules in the database. This should decrease utilization and radiation exposure.

The National Lung Cancer Screening Trial suggested that 24% of CT scans identified nodules^[9]^. We found an average of 64% of CT scans done at our facility identified lung nodules. Local geographic factors including a high incidence of smoking and endemic histoplasmosis may well explain this variance. Kentucky is noted to have the highest incidence of lung cancers in the United States linked to its tobacco and coal heritage. Additionally, our veteran population has a high incidence of heavy smoking and for potential chemical exposures associated with the Vietnam and Gulf Wars.

Beginning in 2008, the LNET program initially received an average of 20 consultations monthly. This did not correlate with the known incidence of lung nodules and put into question the management these nodules. Implementing a direct consultation system increased consultations to 203 per month, representing a more accurate picture of the total incidence of lung nodules at our facility. Previously, many of these were being managed per radiology recommendations and had inconsistent surveillance measures. While radiology recommendations may follow a variety of accepted guidelines, this can result in more frequent radiologic examinations or increased risk with diagnostic interventions. The Fleischner Society released recommendations in 2005 that have been widely adopted, however limitations have prevented universal acceptance^[10]^. Several studies have shown high variability in radiologist recommendations for subsequent follow up and management^[11, 12]^. Our program ensures a specialist in thoracic and lung disease reviews films and assigns a patient to the appropriate management pathway. We use these protocols as guidelines and defer follow-up to professional clinical judgement.

This is a limited retrospective review of our experience with a multidisciplinary approach to the evaluation of lung nodules. An automatic consult to LNET from radiology seems to capture a more accurate picture of the incidence of nodules in this population. A dedicated nurse navigator facilitates the process and assists in ensuring completion of follow up and/or diagnostic and therapeutic interventions. The culmination of this process may give the ability to diagnose malignant disease at an earlier stage when the opportunities for potential cure are the highest.
